# Migration and Spontaneous Extrusion of a Lumbar Spinal Fusion Rod From the Gluteal Region

**DOI:** 10.7759/cureus.20550

**Published:** 2021-12-20

**Authors:** Tamara L Soh, Cheryl M Tan, Kelvin K Lor, Jacob Y Oh

**Affiliations:** 1 Orthopaedic Surgery, Tan Tock Seng Hospital, Singapore, SGP; 2 Orthopaedic Surgery, Khoo Teck Puat Hospital, Singapore, SGP

**Keywords:** spinal instrumentation, implant failure, pseudoarthrosis, scoliosis, adult spinal deformity

## Abstract

Instrumented fusion with rods and pedicle screws is often performed for the surgical treatment of adult spinal deformity (ASD). One of the complications of such long construct fusions is that of pseudoarthrosis, which can present with implant loosening, failure, and rod breakage. However, migration and spontaneous extrusion of the rod is relatively rare and has yet to be reported in the literature. We report a gentleman with previous long construct instrumented fusion done six years ago for ASD, who presented with gluteal pain. Radiographs revealed rod breakage and caudal^ ^migration towards the left gluteal region. He subsequently reported spontaneous extrusion of the broken rod through the gluteal skin, without the need for surgical removal. This case is reported for its rarity and to raise awareness about the rare occurrence of rod migration after breakage that can lead to potential complications if left unattended.

## Introduction

Adult spinal deformity (ASD) is of increasing concern as our population ages and life expectancy increases, along with improved access to healthcare resources. ASD may result in significant physical disability, with adverse effects on one’s mental health, self-rated quality of life, and health status as a whole [1]. In the surgical treatment of ASD, pedicle screws and rods are commonly used to maintain the correction of sagittal and coronal alignment, and provide stability for fusion. A rare sequelae following pseudoarthrosis and implant failure is rod migration. Although there have been cases of caudal rod migration to areas ranging from the gluteal region to the knee described in the literature, these cases required removal of the migrated rods surgically. We describe a case of a patient with migration of a spinal fusion rod and spontaneous extrusion, which is to our knowledge the first in the literature. 

## Case presentation

A 73-year-old gentleman underwent staged lateral lumbar interbody fusion (LLIF) from L2 to L4, followed by posterior instrumentation from T10 to the ilium with L2-L5 posterolateral interbody fusion and L5/S1 transforaminal interbody fusion six years prior. He developed proximal junctional failure of the T10 pedicle screws 10 months later and underwent revision instrumentation from T5 to the ilium. Postoperative recovery was uneventful and subsequent radiographs showed good restoration of alignment.

**Figure 1:**
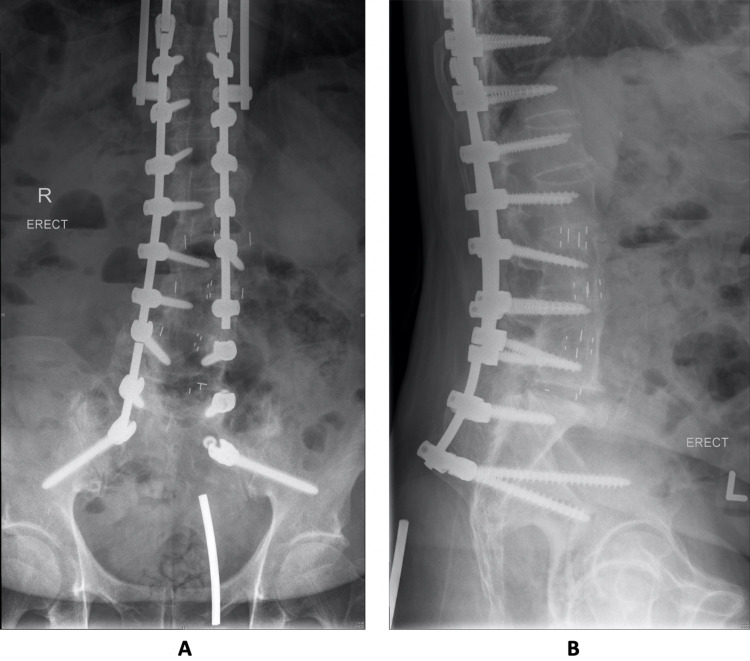
A: AP radiograph of the lumbar spine demonstrating broken rods bilaterally with migration of the rod on the left. B: Lateral radiograph of the lumbar spine demonstrating caudal migration of the rod into the soft tissue. AP, anterior-posterior

However, he returned to the outpatient department reporting acute left buttock pain for one month with pain on sitting, as well as implant prominence over the left gluteal region. On examination, there were no swelling or skin changes over the left gluteal region. However, there was prominence of the rod on deep palpation as well as tenderness over the left gluteal region. Lumbar spine radiographs showed broken rods bilaterally with caudal migration of the rod on the left (Figure 1A,B).

**Figure 2:**
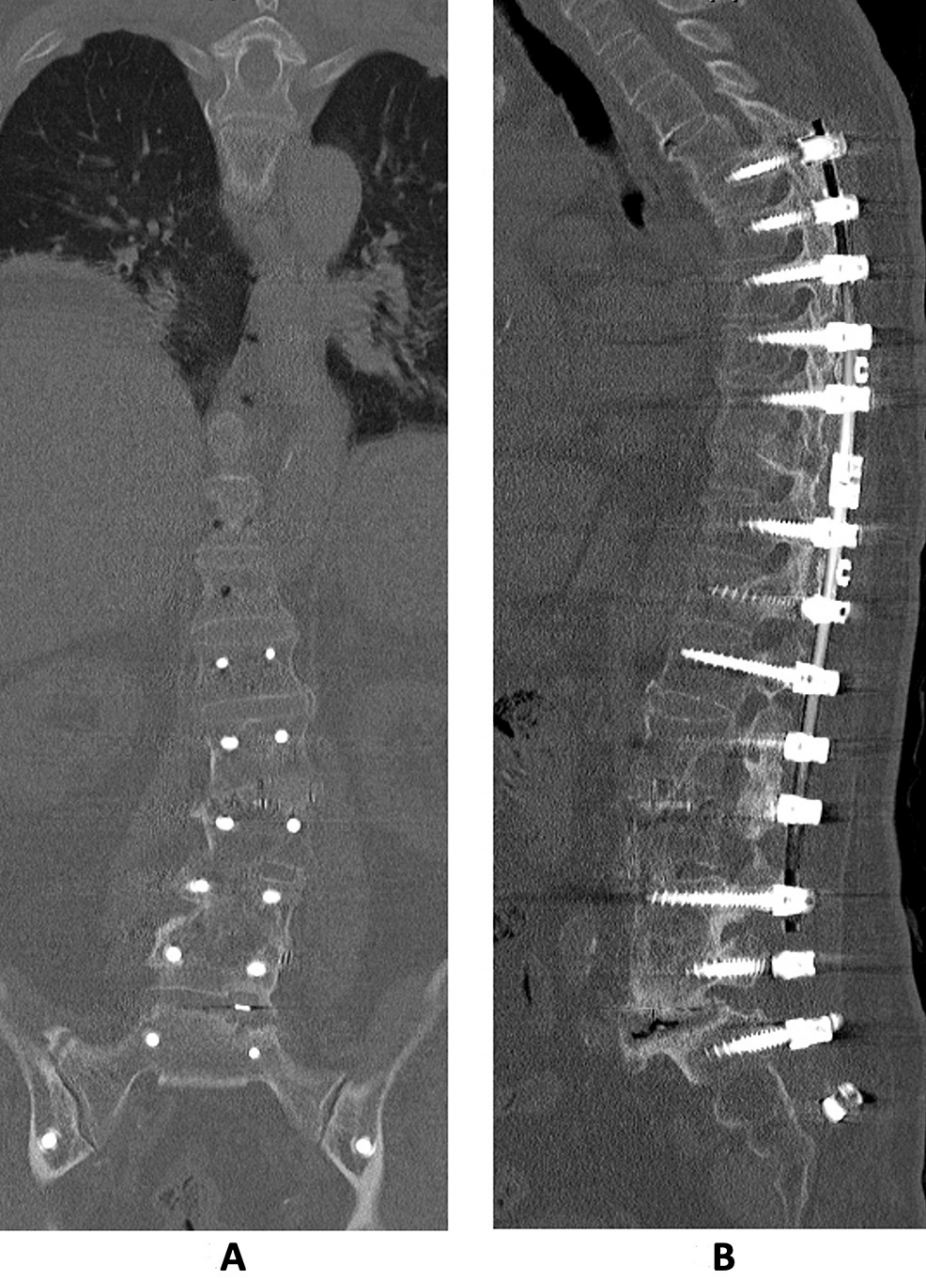
A: Coronal CT images demonstrating pseudoarthrosis at L5/S1 and absence of the broken rod on the left. B: Sagittal CT image showing pseudoarthrosis at L5/S1.

**Figure 3:**
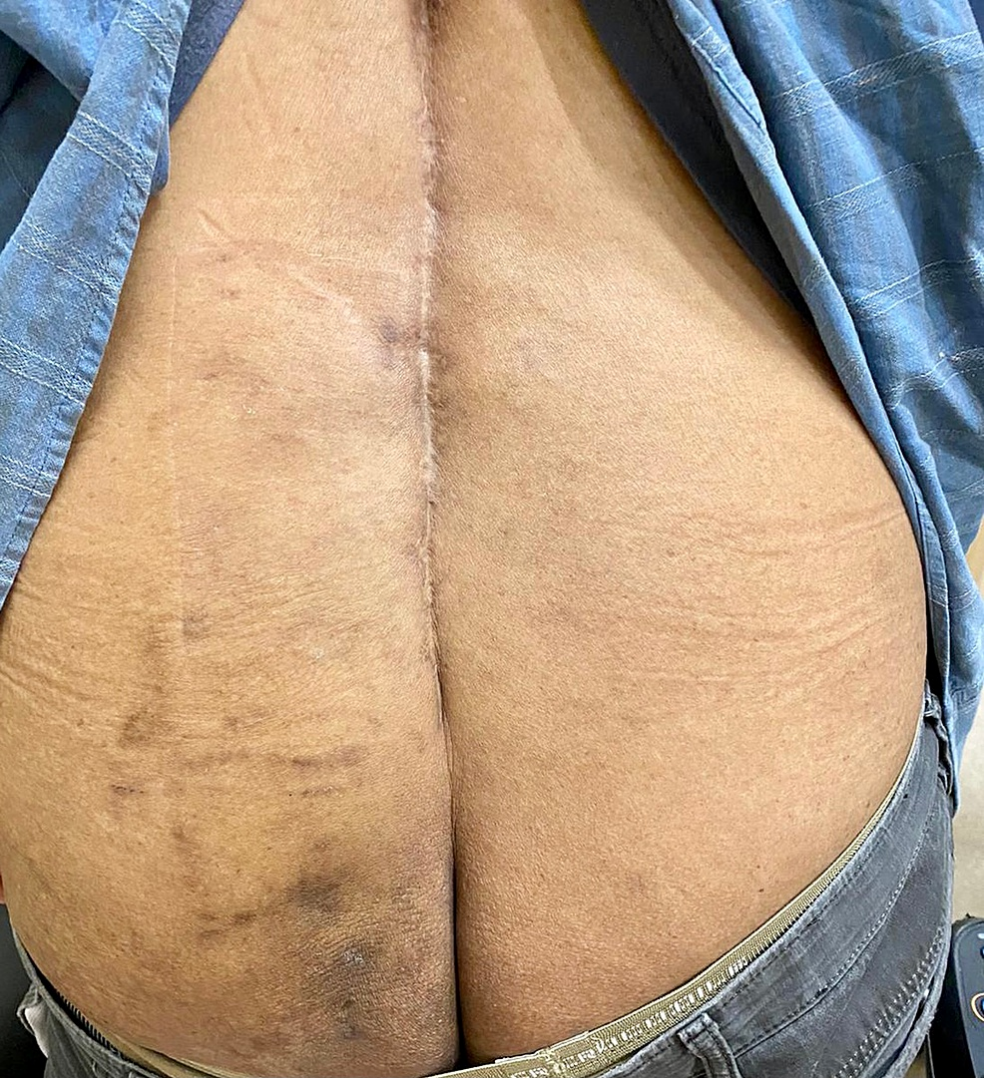
Clinical photograph demonstrating a healed wound over the left gluteal region from the extruded rod.

**Figure 4:**
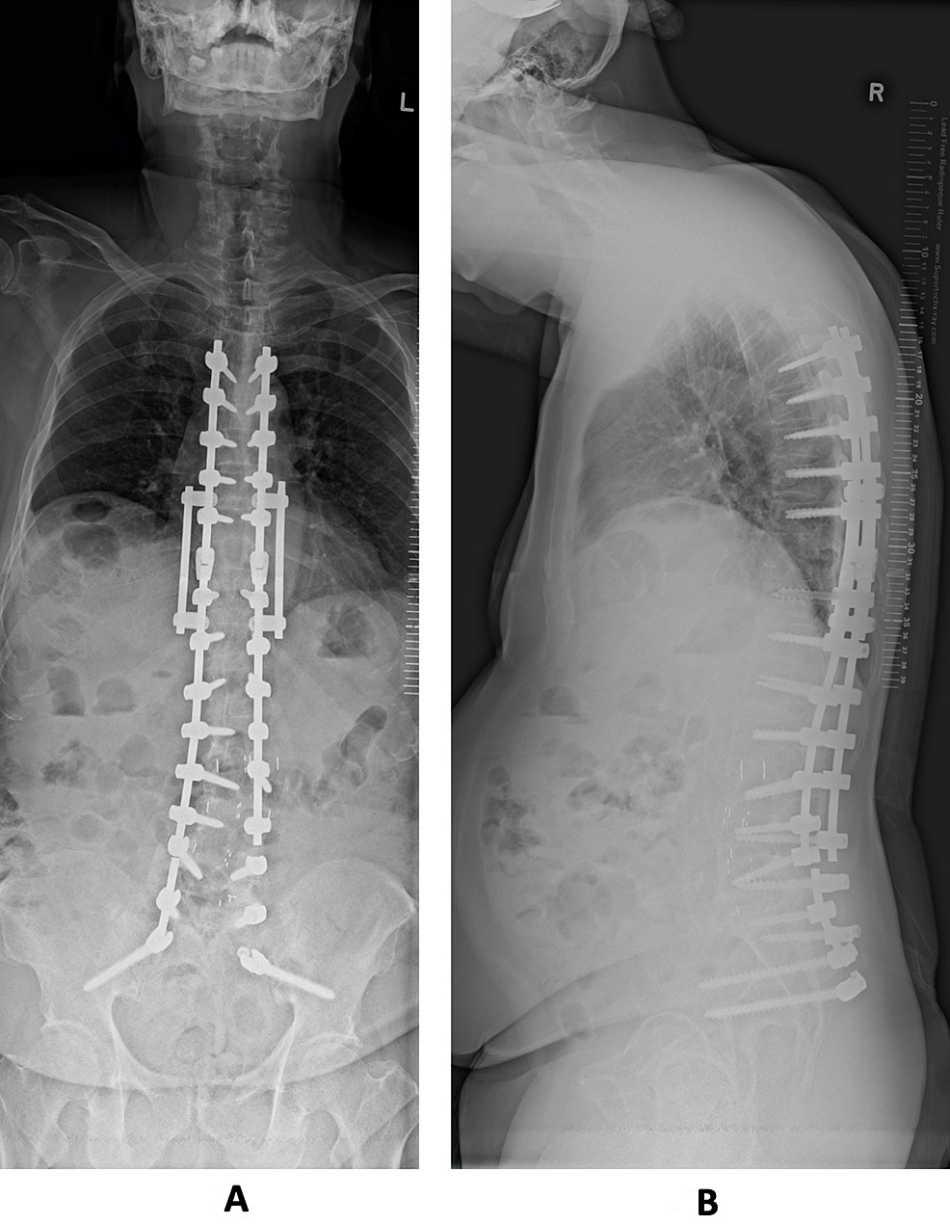
A: AP of the spine demonstrating absence of the broken rod on the left, and a broken rod on the right below the L5 screw. B: Lateral radiograph of the spine showing the absence of the broken rod on one side, with the contralateral rod broken but not migrated. AP, anterior-posterior

A CT scan was arranged which demonstrated pseudoarthrosis at L5/S1, and the absence of the rod on the left (Figure 2A,B). At the repeat follow-up, the patient reported that there was the formation of a sinus from the implant prominence, which led to spontaneous extrusion of the rod from his gluteal region while he was sitting on the toilet. This occurred just prior to his CT scan, which resulted in the resolution of his implant prominence-related symptoms. He had no further back pain or radiculopathy symptoms. Physical examination revealed a healed wound over the left gluteal region (Figure 3). Repeat lumbar radiographs confirmed broken rods bilaterally, with the absence of the broken and migrated rod on the left side (Figure 4A,B). As he had resolution of his symptoms, and CT scan demonstrated good fusion across the other instrumented levels, the L5/S1 pseudoarthrosis was managed non-operatively. 

## Discussion

Implant-related complications are widely recognized to be a leading cause for revision surgery in the treatment of ASD. Rod fracture has been reported to be the most common of implant-related complications [2], with an incidence ranging from 6.8% to 18.4% in the literature [3-4]. This is usually, although not always, related to pseudoarthrosis, in which there is cyclical loading and eventual fatigue failure of the rods, leading to loss of correction and pain.

Prior authors have identified risk factors for rod fracture in ASD, such as a greater preoperative sagittal imbalance, a greater preoperative thoracolumbar kyphosis, and the use of smaller diameter rods [4]. In our patient, the pseudoarthrosis at L5/S1 most likely contributed to an increased risk of rod fracture. However, despite the rod fracture, the sagittal and coronal correction was maintained and he remained asymptomatic apart from the episode when the rod was extruded spontaneously. In a review of outcomes after rod fracture, the authors reported that a bilateral rod fracture was more likely to represent non-union, while a unilateral rod fracture may or may not represent non-union. This in turn affects outcomes, as patients with bilateral rod fractures, compared to those with unilateral rod fractures, were found to have worse patient-reported outcomes and pain scores after surgery [4].

In the case presented, the rod fracture was accompanied by loss of fixation to the pedicle screwhead, resulting in distal migration of the rod. Rod migration, although extremely rare, has also been reported, with instances of rods migrating to regions ranging from the knee to the pelvis [5-6]. This is of no trivial matter, as rod migration can result in adverse outcomes such as visceral, vascular, or neurological injury [7]. We hypothesize that the distal migration into the soft tissue of the gluteal region may not have been symptomatic initially due to an abundant soft tissue envelope, whereas in contrast, a broken pedicle screw with loosening and backing out may result in implant prominence over the back early on. However, the exact soft tissue track of the migrated rod is not known and the patient was fortunate to not have developed skin or soft tissue complications such as a chronic or infected sinus tract formation. In a literature review of rod migration by Bayri et al. [8], all cases necessitated surgical intervention for rod removal, apart from those cases where the patients declined surgery. Our case is unique and the first in the literature to have extruded from the soft tissue spontaneously, without the need for surgical removal.

## Conclusions

Although rod breakage may be a relatively common complication of ASD surgery, resultant sequelae such as rod migration remain relatively rare. However, it is important to be vigilant in the follow-up of patients undergoing ASD surgery, as rod breakage and migration due to pseudoarthrosis can lead to serious neurological, visceral, and vascular sequelae. We present the first known case of caudal rod migration with spontaneous extrusion from the skin in a patient with previous long-construct instrumented fusion.
